# Obtaining Bioactive Compounds from the Coffee Husk (*Coffea arabica* L.) Using Different Extraction Methods

**DOI:** 10.3390/molecules26010046

**Published:** 2020-12-24

**Authors:** Mariana de Oliveira Silva, John Nonvignon Bossis Honfoga, Lorena Lucena de Medeiros, Marta Suely Madruga, Taliana Kênia Alencar Bezerra

**Affiliations:** Technology Centre, Department of Food Engineering, Federal University of Paraiba, Joao Pessoa 58051-900, Brazil; marianaoliveira.ufpb@gmail.com (M.d.O.S.); jnbossis91@hotmail.com (J.N.B.H.); lorenalucena@live.com (L.L.d.M.); msmadruga@uol.com.br (M.S.M.)

**Keywords:** phenolic compounds, antioxidant capacity, plant extract, coffee husk, conventional extraction, ultrasound-assisted extraction

## Abstract

Coffee husks (*Coffea arabica* L.) are characterized by exhibiting secondary metabolites such as phenolic compounds, which can be used as raw material for obtaining bioactive compounds of interest in food. The objective of this study is to evaluate different methods for obtaining the raw material and extracting solutions of bioactive compounds from coffee husks. Water bath and ultrasound-assisted extraction methods were used, using water (100%) or ethanol (100%) or a mixture of both (1:1) as extracting solutions and the form of the raw material was in natura and dehydrated. The extracts were evaluated by their antioxidant potential using DPPH radicals, ABTS, and iron reduction (ferric reducing antioxidant power (FRAP)), and later total phenolic compounds, total flavonoids, and condensed tannins were quantified the phenolic majority compounds were identified. It was verified that the mixture of water and ethanol (1:1) showed better extraction capacity of the compounds with antioxidant activity and that both conventional (water bath) or unconventional (ultrasound) methods showed satisfactory results. Finally, a satisfactory amount of bioactive compounds was observed in evaluating the chemical composition (total phenolic compounds, total flavonoids, condensed tannins, as well as the analysis of the phenolic profile) of these extracts. Corroborating with the results of the antioxidant activities, the best extracting solution was generally the water and ethanol mixture (1:1) using a dehydrated husk and water bath as the best method, presenting higher levels of the bioactive compounds in question, with an emphasis on chlorogenic acid. Thus, it can be concluded that the use of coffee husk as raw material to obtain extracts of bioactive compounds is promising. Last, the conventional method (water bath) and the water and ethanol mixture (1:1) stood out among the methods and extracting solutions used for the dehydrated coffee husk.

## 1. Introduction

Brazil is considered the world’s largest producer and exporter of coffee, and is also the second largest consumer of the product. The country accounts for 30% of the international market, producing a total of approximately 20 million bags, with 46.25% for domestic consumption [1,2,3].

Coffee processing can be done by dry or wet routes, generating different types of residues [4]. According to Setter et al. [5], the most used method in Brazil is dry processing, with the main residue generated being the husk obtained with a yield of approximately 50% of the weight of the coffee beans. Coffee husks are characterized by secondary metabolites, such as caffeine and tannins, and phenolic acids such as chlorogenic acid, thereby being a potential source for obtaining bioactive compounds of interest to apply in food. Chlorogenic acid is widely distributed in the plant kingdom, and coffee and its by-products are considered its greatest source. The biological function of this phenolic compound is linked to its antioxidant, antimicrobial, and anticarcinogenic activities [6,7,8].

Bioactive compounds are widely studied and widely found in plant sources, in particular phenolic compounds, which can have numerous applications in science and the food industry [9]. According to García and Bianchi [10], phenolic compounds constitute one of the main classes of antioxidants from natural sources, being distributed in leaves, seeds, grains, roots, and bark. In addition to the antioxidant activity as technological application, the presence of these compounds has been attributed to benefits in human health by delaying or preventing the appearance of diseases due to the antioxidant action in the organism, as well as anti-inflammatory, antimicrobial, and anti-allergic actions.

The extraction procedure is the most important step for recovering phenolic compounds from vegetables, directly affecting the yield of the bioactive compounds [11,12]. According to Wijngaard et al. [13], different techniques are used to extract these compounds, as determined by conventional and non-conventional methods.

Ultrasound-assisted extraction (UE) is a non-conventional method which consists in applying mechanical waves that aim to intensify the extraction by means of a phenomenon called cavitation. This is an indirect method, where the waves are propagated through the sample container while the probe acts directly on the matrix and solvent [14,15]. However, conventional processes, such as extraction using water bath, are based on the use of solvents that extract the compounds of interest from the solute or matrix, being associated with the use of heat [16].

The recovery of bioactive compounds from different plant matrices, such as jaboticaba peel, grape pomaces, red araçá peel, silver, and coffee grounds, has already been reported in several studies. In this research, different extraction methods were used, such as conventional extraction by maceration and heat application as well as ultrasound-assisted extraction [12,16,17,18,19].

In light of the above, the objective of this study was to evaluate different extraction methods—water bath (B) and ultrasound (U)—of bioactive compounds from coffee husks (*Coffea arabica* L.) in natura and after dehydration (D) using different extracting solutions—ethanol €, water (W), and mixture ethanol: water (M)—and evaluating the antioxidant activity of the extracts.

## 2. Results and Discussion

### 2.1. Effects and Interactions of Extraction Variables on the Antioxidant Activity of Coffee Husk Extracts (Coffea arabica L.)

**Figure 1:**
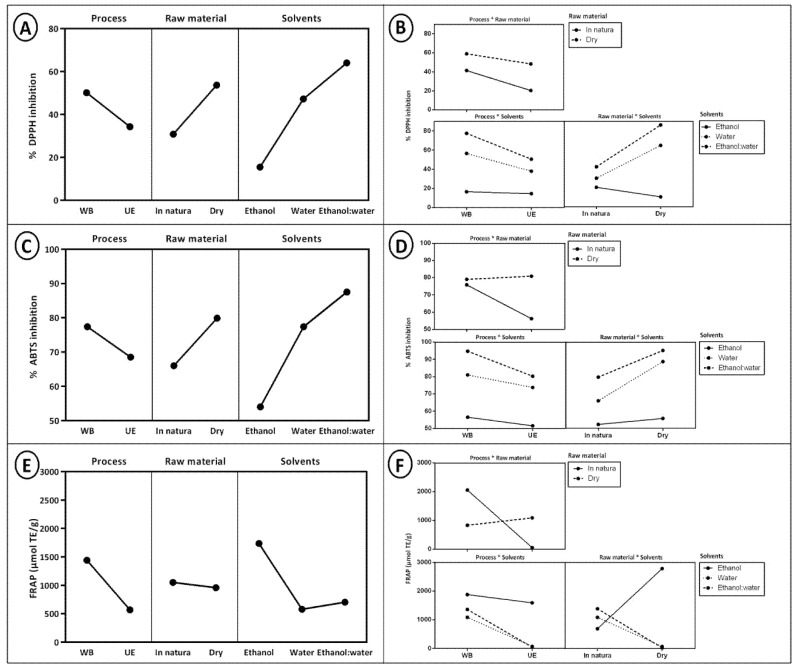
(**A**) Effects of extraction variables for DPPH; (**B**) Effects of interaction between extraction variables for DPPH; (**C**) Effects of extraction variables for ABTS; (**D**) Effects of interaction between extraction variables for ABTS; (**E**) Effects of extraction variables for ferric reducing antioxidant power (FRAP); (**F**) Effects of interaction between extraction variables for FRAP.

Table 1 presents the mean values referring to the antioxidant activity (DPPH, ABTS, and ferric reducing antioxidant power (FRAP)) of the extracts, and Figure 1 shows the effects of the extraction variables on the study responses. The extracts were obtained from conventional (water bath) and non-conventional (ultrasound-assisted) extraction methods of the coffee husk using different extraction systems—water (100%), ethanol (100%), or its mixture (1:1)—as an extraction solution. Thus, the entire study was performed on both in natura and dehydrated husks.

The extraction methods were called processes, with the solvent extraction solutions and the way to obtain the coffee husk as raw material. The interaction between process and raw material (*p* = 0.0001), process and solvent (*p* < 0.0001), and raw material and solvent (*p* < 0.0001) had a significant effect on the antioxidant activity measured by the DPPH method. The extraction in water bath, the use of dehydrated husk, and the water and ethanol mixture (1:1) presented higher efficiency, and consequently obtained extracts with a high inhibition percentage of DPPH (84.95%), as shown in Figure 1A,B.

According to Thouri et al. [20], the polarity of the solvent used for extraction has a great influence on the level of polyphenols extracted. The optimal extraction of the polyphenols is obtained in polar solvents because they present better efficiency in the solvation process as a result of the interaction (hydrogen bridges) between the polar sites of the antioxidant compounds and the solvent.

Therefore, polar solvent mixtures are used for recovering phenolic compounds. Water is a highly polar solvent, while ethanol has less polarity; however, both solvents can be mixed, forming a more efficient combination to extract antioxidant compounds [21]. It is also observed that dehydrating the sample provided greater concentration of bioactive compounds in the extracts and thus intensified the antioxidant action. Pereira et al. [22] report that the rupture of the raw material structure during the dehydration and drying process promotes a release of more phenolic compounds.

The values found in this test were superior (260 times) to those found by García and Bianchi [10], who presented 23.1% in acetonic extract of the dehydrated husk of robusta coffee. Variations in antioxidant activity values found in the literature may be associated to the variety of the raw material, solvents, and extraction methods used [23].

The interactions between process and raw material (*p* < 0.0001), process and solvent (*p* = 0.03), and raw material and solvent (*p* < 0.0001) presented significant effects in evaluating the antioxidant action against the ABTS radical (Figure 1C,D). In the same way as the DPPH radical, the water bath extraction, the use of the dehydrated husk, and the action of the water and ethanol mixture (1:1) showed prominence for extracting bioactive compounds which provide antioxidant action against this radical (92.81%).

However, water (100%) is also highlighted for the ABTS in addition to the water and ethanol mixture as an extracting solution. The literature reports that the use of organic solvents such as ethanol mixed with water favors creating a polar environment, facilitating the extraction of polyphenols from coffee husks. However, the use of water as the only extraction solvent provides the extract with impurities such as organic acids and sugars, which can interfere with phenolic quantification [24,25].

Andrade et al. [6] studied the extraction of bioactive compounds from coffee residues (spent coffee grounds and coffee husks) using the ultrasound method and ethanol as an extracting solution. These authors found lower values than in this work of 13.3% for the husk and 10.6% for lees, which can be justified by the use of a different extracting solution (ethanol).

According to Figure 1E,F, it is possible to observe different behavior of the extracts with respect to FRAP. The highest antioxidant activity for the DPPH and ABTS methods was measured by the dehydrated coffee husk together with a water and ethanol mixture (1:1). The highest antioxidant activity for the FRAP method was found in the extract which used the coffee husk in natura and ethanol as solvent (100%). The interaction between process and raw material (*p* < 0.000), process and solvent (*p* < 0.000), and raw material and solvent (*p* < 0.000) had a significant effect on the antioxidant activity measured for this method. The extracts with the highest capacity to reduce the iron ion for conventional water bath extraction were those that used the in natura husk with the ethanol:water (1:1) (2639.4 μmol TE/g,) and ethanol (100%) (1291.85 μmol TE/g) extract solutions. However, the extract which used ethanol extract solution (100%) but with the dehydrated husk also showed satisfactory values (2425.9 μmol TE/g). Moreover, the extract with the highest capacity to reduce the iron ion in the ultrasound-assisted extraction was the extract obtained with the ethanol extract solution (100%) using the dehydrated coffee (3136.4 μmol TE/g).

Measuring FRAP antioxidant activity consists of the capacity of the antioxidant compounds to reduce Fe^3+^ to Fe^2+^, with this reaction being performed in an interval of 30 min [26]. According to Barros et al. [12], this difference in behavior between the methods can be explained by the fact that few phenolic compounds, such as quercetin, cannot reduce Fe^3+^ within 30 min, thus it does not enable its measurement within this interval, requiring a greater reaction time than 30 min for its total quantification, in addition to the fact that the reduction of iron may occur more quickly or does not depend on the type of solvent. Therefore, these two factors may explain why ethanol presented higher antioxidant activity than the other solvents.

Muñoz et al. [27] evaluated the antioxidant capacity of green and roasted coffee by FRAP analysis and found values of 21.04 μmol TE/g and 23.25 μmol TE/g, respectively. Barros et al. [12] found a value of 722 μmol TE/g in jabuticaba bark extract, where the extraction of bioactive compounds was also performed with ultrasound and the mixture of solvents water and ethanol as an extraction solution.

Therefore, it can generally be seen that the mixture of ethanol and water solvents proved to be the most efficient solvent for extracting phenolic compounds from the coffee husks in this study. According to Mokrani and Madane [24], the solubility of these compounds mainly depends on the polarity of the solvent chosen to extract them, its polymerization degree, the interaction with other food components, and the formation of insoluble complexes.

In addition to the use of the mixture, differences between the two extraction methods were also observed. The extracts that were obtained through conventional water bath extraction showed higher antioxidant activity compared to those by ultrasound-assisted extraction. However, extracts obtained by ultrasound also presented satisfactory values in the ABTS and FRAP analyses. According to Meregalli et al. [16], the use of conventional water bath extraction is a more advantageous process compared to ultrasound-assisted extraction, as the equipment is of high value. Therefore, it can be observed that the conventional extraction used in this study could better obtain extracts which were rich in bioactive compounds with high antioxidant activity from the coffee husks; thus, presenting an alternative use for the coffee husk, minimizing the amount of agro-industrial residue generated by the coffee industry and adding value to it.

Another relevant factor was the influence of the moisture content of the raw material, i.e., the use of the husk in natura (82.74% ± 0.60) or dehydrated (10.49% ± 0.13). It is verified that the most satisfactory results were presented by the extracts which used the dehydrated raw material. The drying process made the compounds present in the coffee husk more concentrated when the water content was removed. However, Stepien et al. [28] report that ideal conditions are needed in the drying process so that there are no adverse changes in the chemical composition of the raw material and that its bioactive properties are preserved.

### 2.2. Determination of Bioactive Compounds

**Figure 2:**
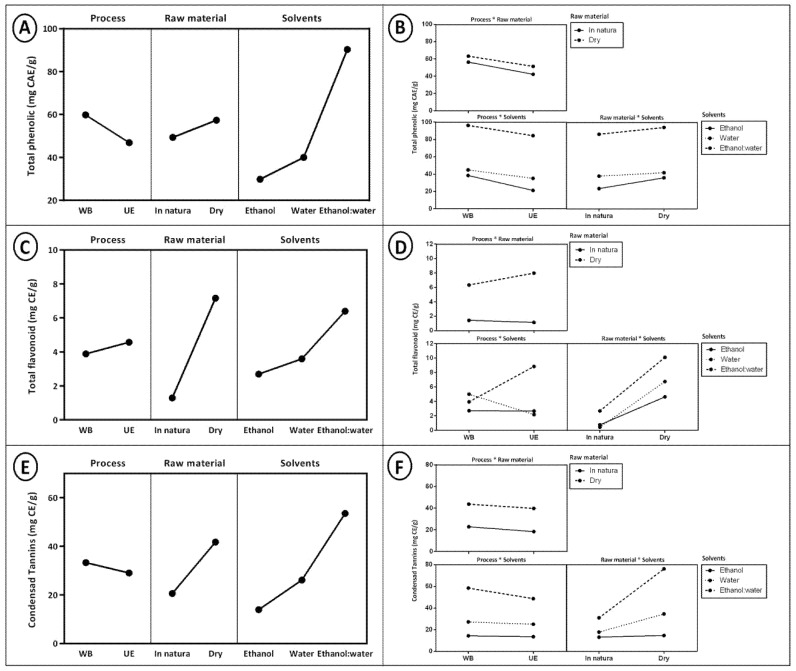
(**A**) Effects of extraction variables for total phenolic. (**B**) Effects of interaction between extraction variables for total phenolic. (**C**) Effects of extraction variables for flavonoids. (**D**) Effects of interaction between extraction variables for flavonoids. (**E**) Effects of extraction variables condensed tannins. (**F**) Effects of interaction between extraction variables for condensed tannins.

Table 2 shows the average values of total phenolic, total flavonoid, and condensed tannin content of coffee husk extracts, and Figure 2 shows the effects of extraction variables on the responses.

According to Figure 2A,B, the statistical analysis showed that the interaction between process and raw material (*p* = 0.001), process and solvent (*p* < 0.000), and raw material and solvent (*p* < 0.000) had a significant effect on total phenolic content. The extracts obtained by the conventional extraction method (water bath) were highlighted in the phenolic values (31.35–97.89 mg CAE/g). Among these extracts, those which were elaborated with the water and ethanol mixture (1:1) showed a higher value of 97.89 mg CAE/g, as shown in Table 2. However, the extract obtained with ultrasound also showed satisfactory results of 90.95 mg CAE/g using the same extracting solution and both with dehydrated husks. Thus, it can be observed that dehydration was a very important factor to concentrate and provide higher phenolic compound levels, while the extracting solution with higher potential was the water:ethanol mixture (1:1).

The antioxidant properties of the coffee husk are a consequence of the presence of caffeine and chlorogenic acid, with these two being the bioactive compounds found in greater abundance in coffee husks [6]. Chlorogenic acid belongs to the family of esters formed by hydroxynamic acids, caffeic acid together with quinic acid, while caffeine is found in several beverages of economic and cultural importance such as coffee, tea, and soft drinks [29]. Andrade et al. [6] studied coffee residues and found the presence of total phenolic compounds ranging from 16.1 mg CAE/g to 151 mg CAE/g for husk and from 24 mg CAE/g to 587.7 mg CAE/g for spent coffee grounds using different extracting solutions and extraction techniques.

Regarding the total flavonoid analysis, according to Figure 2C,D it can be observed that the statistics indicate that the process (*p* = 0.01), the raw material (*p* < 0.000), and the solvents (*p* < 0.000) had a significant effect. In agreement with the other analyses of the study, the use of the dehydrated husk was more efficient for extracting bioactive compounds from the coffee husk. In relation to the conventional extraction (water bath), the extract obtained with water (100%) and that used the dehydrated coffee husk presented a more expressive result in the flavonoid content (9.93 mg CE/g), followed by the extract obtained with the water and ethanol mixture (1:1) (4.53 mg CE/g). The extract which presented the best result in the ultrasound-assisted extraction used the solvent mixture (15.69 mg CE/g).

The prominence of water as an extracting solution is attributed to the polarity of the solvent and its affinity for extracting this type of phenolic compound. According to Mokrani and Madane [24], there is not a single solvent capable of extracting all classes of phenolic compounds from a sample simultaneously, so there are solvents which extract one type of compound better than another.

Therefore, the evaluation regarding the extracting solutions is very important, because the solubility of the phenolic compounds greatly depends on the chemical nature of the sample and the polarity of the solvent used to extract them, as the quantity of these compounds found in food is directly related to its antioxidant capacity [12]. Saada et al. [30] found total flavonoid values of 2.58 mg CE/g using ethanol (100%) and 2.8 mg CE/g using an ethanol:water mixture (1:1), both by conventional extraction by maceration of the coffee silverskin and coffee ground by-products, respectively.

In evaluating the condensed tannins, according to Figure 2E,F it can be observed that the statistical analysis showed that the process (*p* < 0.000), raw material (*p* < 0.000), and solvents (*p* < 0.000) presented significant effects. Furthermore, the use of dehydrated husk and the water and ethanol mixture (1:1) presented greater efficiency with the water bath extraction (79.71 mg CE/g).

Condensed tannins are formed by chains of catechin, epicatechin, and the esters of gallic acid, which may be associated with potential health benefits [8]. The values found in this study were higher than those highlighted by Saada et al. [30], who found condensed tannin values in coffee silverskin and coffee ground by-products of 3.84 mg CE/g and 0.997 mg CE/g, respectively, using conventional extraction by maceration. Castaldo et al. [31] reports that tannins are mainly found in coffee pulp and husks.

### 2.3. Profile of Phenolic Compounds

The major phenolic compounds which were found in coffee husk extracts are presented in Table 3, and the results are expressed in µg/g. The phenolic compound identified in higher concentration in both extracts was chlorogenic acid, ranging from 16.64 to 337.07 µg/g. Gallic and caffeic acids were also identified, however, in lower concentrations. The phenolic compounds which represent the main phenolic amount in coffee beans and which are most discussed in the literature are chlorogenic acids and their metabolites [6,7]. Thus, several studies involving coffee beans and their by-products have identified and quantified phenolic acids such as chlorogenic, gallic, and caffeic acids [23,27,31,32] in these matrices, being in accordance with the compounds identified in this study.

The best way to extract these compounds was by the conventional method (water bath) using the dehydrated husk and ethanol:water solvent mixture (1:1), being in accordance with the results of total phenolic compounds for which the same extract obtained the best result in the recovery of the compounds. However, extracts that used water as a solvent (100%) by both the conventional method and by ultrasound-assisted extraction were also able to recover amounts of chlorogenic, gallic, and caffeic acid.

### 2.4. Principal Component Analysis (PCA)

**Figure 3:**
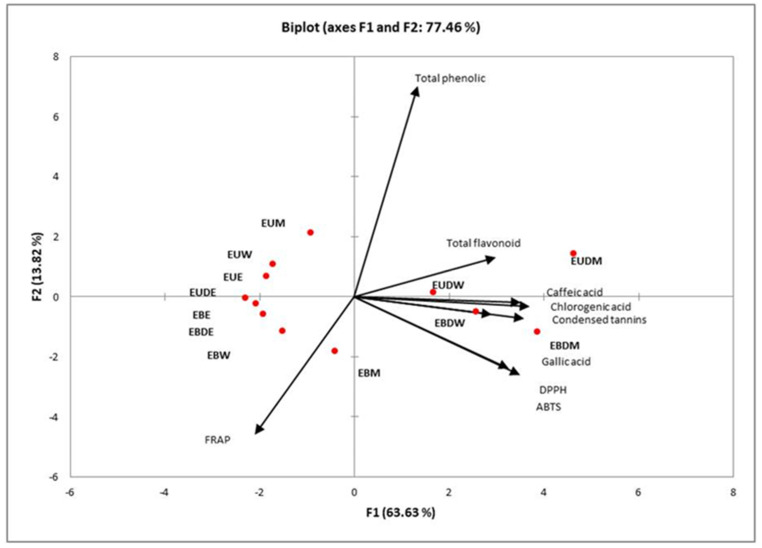
Principal component analysis (PCA).

The analysis of principal components was applied to generally evaluate the behavior of the extraction methods, the extracting solutions, and the dehydration of the coffee husk through the results obtained from the antioxidant activity, total phenolic compounds, total flavonoids, condensed tannins, and the profile of the extracted compounds (Figure 3). The F1 and F2 principal components presented variance of 63.63% and 13.82%, respectively.

According to Figure 3, it is noted that the extracts which presented greater antioxidant action against the DPPH and ABTS radicals were those which used the dehydrated coffee husks, both through the conventional water bath extraction and by ultrasound-assisted using the water and ethanol solvent mixture (1:1) or only water (100%). The total phenolic compounds, total flavonoids, and tannins are observed in the same grouping, as well as the compounds identified in the phenolic profile (chlorogenic, galic, and caffeic acids), indicating the antioxidant action of these compounds present in the extracts. According to Araújo et al. [21], the antioxidant activity of vegetable raw materials is often related to the presence of bioactive compounds. Andrade et al. [6] evaluated the extraction of phenolic compounds in coffee husks and also identified compounds such as chlorogenic acid, gallic acid, and caffeic acid.

## 3. Materials and Methods

### 3.1. Experimental Design

**Figure 4:**
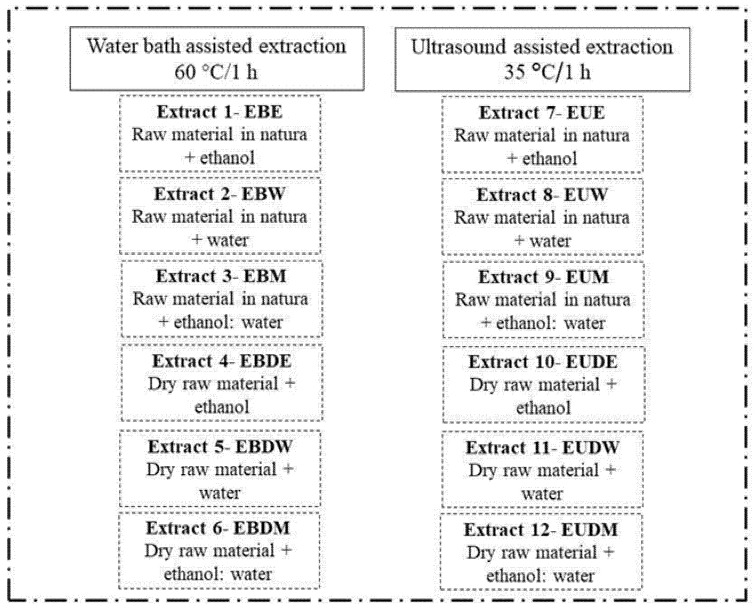
Representative scheme of extraction procedure.

The extraction study of the compounds was carried out using the conventional (water bath) and unconventional (ultrasound) extraction method with three solvent systems (water, ethanol, and the 1:1 mixture), as shown in Figure 4, both in the in natura husk and in the dehydrated husk.

### 3.2. Husk Dehydration

The coffee husks used in the work were supplied by a company that produces 100% arabica and typica coffee, located in the city of Taquaritinga do Norte, PE, Brazil (Latitude: −7.88809, 36°5′33″ West). The raw material was used both in its fresh form with a moisture content of 82.74 ± 0.60% and after going through an oven drying process with forced air circulation at 40 °C for 48 h and then ground in a ball mill (Solab, São Paulo, Brazil), presenting a final moisture content of 10.49 ± 0.13%.

### 3.3. Extraction Procedure

The extraction solution (100% water or 100% ethanol or water + 1:1 ethanol) and the extraction methods applied were selected based on studies in the literature [6,29,33,34,35]. The extraction in a water bath was performed following the methodology of Neto [35]. The raw material (in natura husk or dehydrated husk) was homogenized manually for 5 min with the extracting solution 1:10 (*p*:*v*), subjected to incubation in a water bath (Cientec, CT 245-9, São Paulo, Brazil) at 60 °C for 1 h. Then, the mixture was centrifuged at 3500× *g* for 20 min at 10 °C (Solab, SL 706, São Paulo, Brazil), and the supernatants were collected and filtered on qualitative filter paper (80 g·m^−2^).

Ultrasound extraction (UE) was performed following the methodology of Andrade et al. [6], with adaptations. The extraction was carried out for 1 h with an extractor solution of 1:10 (*p*:*v*) at a temperature of ±35 °C. Then, the mixture was centrifuged at 3500× *g* for 20 min at 10 °C (Solab, SL 706, São Paulo, Brazil), and the supernatants were collected and filtered on qualitative filter paper (80 g·m^−2^). The equipment used was an ultrasonic bath (Marconi, São Paulo, Brazil), operating at a frequency of 40 kHz and power of 220 V.

### 3.4. Antioxidant Activity

#### 3.4.1. Sequestering Activity for the 2,2-Diphenyl-1-Picrylhydrazyl Radical (DPPH•)

The ability of coffee husk extracts to sequester the DPPH• radical was determined according to the method described by Brand-Williams, Cuvelier, and Berset [36]. The radical elimination activity according to the antioxidant capacity of the extracts coffee husk was verified at 515 nm in a UV–Vis spectrophotometer (Quimis, São Paulo, Brazil) in triplicate. The antioxidant potential of the samples was expressed as the percent inhibition of the DPPH• radical, according to Equation (1).

(1) $$\%Inhibition=\left[\frac{\left(ADPPH-Aextract\right)}{ADPPH}\right]\times \;100$$

#### 3.4.2. Sequestering Activity for the 2,2-Azino-bis (3-Ethylbeothiazoline)-6-Sulphonic Acid Radical (ABTS•+)

The ability to sequester the ABTS•+ radical was determined according to the method proposed by Re et al. [37]. The radical elimination activity was verified according to the antioxidant capacity of the coffee husk extracts at 734 nm in a UV–Vis spectrophotometer (Quimis, São Paulo, Brazil). The antioxidant potential of the samples was expressed as the percentage of inhibition of the ABTS•+ radical, according to Equation (2)**.**

(2) $$\%\;Inhibition=\left[\frac{\left(Abs.ABTS-Aextract\right)}{Abs.ABTS}\right]\;\times \;100$$

#### 3.4.3. Ferric Reducing Antioxidant Power (FRAP)

The ferric reducing capacity was evaluated using the ferric reducing antioxidant power (FRAP) method described by Benzie and Strain [26]. According to the antioxidant potential, the ability of the coffee husk extracts to reduce iron (Fe^3+^) to the ferrous form (Fe^2+^) was verified at 593 nm in a UV-VIS spectrophotometer (Quimis, São Paulo, Brazil). Based on the calibration curve prepared with different concentrations of Trolox (50–1000 µM), the results were expressed as the equivalent of μmol of Trolox/g of sample.

#### 3.4.4. Total Phenolic Content (TPC)

The content of total phenolic compounds was determined to the according Folin-Ciocalteau method proposed by Singleton and Rossi [38], adapted by Andrade et al. [6]. Briefly, the eaction mixture was composed by 0.1 mL of extract, 7.9 mL of distilled water, 0.5 of Folin-Ciocalteau reagent and 1.5 mL of 20% sodium carbonate. The flasks were agitated, held for 2 hand absorbance was mensured at 465 nm. TPC was calculated according to a standard curve, prepared with chlorogenic acid and the results were expressed in milligrams of equivalent chlorogenic acid (CAE) per gram of sample (mg CAE/g).

#### 3.4.5. Total Flavonoid Content

The content of total flavonoids was determined to the according method proposed by Zhishem et al. [39] with some adaptations. The reaction system was carried out through 0.15 mL of NaNO_2_, waiting to react for 5 min, followed by the addition of 0.15 mL of AlCl_3_ and waiting for the reaction to proceed for 6 min. Finally, 1.0 mL of NaOH and 1.2 mL of distilled water were added. The absorbance readings were performed on a spectrophotometer UV-VIS (Quimis, Q798U, São Paulo, Brazil), at 510 nm. The quantification of total flavonoids in the extracts was performed using a standard curve prepared with catechin and expressed in milligrams equivalent of catechin (CE) per gram of sample (mg CE/g).

#### 3.4.6. Condensed Tannins

The content condensed tannins was determined to the according method proposed by Broadhurst & Jone [40]. Aliquot of 30 μL of the extract was mixed with 900 μL of 4% (*w*/*v*) vanillin prepared with methanol and then 450 μL of concentrated HCl was added. The mixture was incubated at room temperature for 20 min under light. The absorbance reading was performed at 500 nm using the UV/Visible spectrophotometer (Quimis, Q798U, São Paulo, Brazil). The quantification was performed using a standard curve prepared with catechin and expressed in milligrams equivalent of catechin (CE) per gram of sample (mg CE/g).

### 3.5. Profile of Phenolic Compounds

The identification and the relative quantification of the phenolic compounds present inthe coffee husk extracts were achievd by a liquid chromatography (HPLC) in reverse phase using a C18 columm (4.6 mm × length 250 mm, 5 µ particle size, Varian, Santa Clara, EUA) and UV/VIS detector. An aliquot of 20 μL of each solution was injected into the HPLC columm maintained at 40 °C using a mobile phase consisting of acetonitrile/0.1% formic acid (15:85, *v*/*v*) flowing at a flow rate of 0.8 mL min^−1^. The quantification was based on external standard method by comparison with the retention time of pure standards of phenolic compounds. For all samples, the final concentration of the compounds was be determined by averaging the results of three consecutive injections [6].

### 3.6. Statistical Analysis

Analysis of the influence of extraction variables (process, obtaining raw material, and extracting solution) on the antioxidant activity and the of content total phenolic compounds and total flavonoids by analysis of variance (ANOVA) using the Minitab 16^®^ software (Minitab Inc., State College, PA, USA) with a 95% confidence level (*p*-value ≤ 0.05). Principal component analysis (PCA) was performed using XLSTAT software version 5.03 (Addinsoft, New York, NY, USA, 2014).

## 4. Conclusions

The use of coffee husk as a raw material for extract obtention is a promising strategy due to the presence of bioactive compounds with antioxidant action. Besides, the use of coffee husks helps to minimize the amount of agro-industrial waste while adding additional value to this specific waste. In this study, the conventional and unconventional methods of extraction were efficient in the process, with the extraction by water bath being better than extraction by ultrasound, which also had the benefit of low cost. The best extracting solution was the water:ethanol (1:1) mixture, showing greater efficiency in the antioxidant compounds recovery from the coffee husk extracts, as well as the use of the dehydrated raw material.

Therefore, future studies on the application of these extracts by the food industry should be carried out, such as the subsequent drying of the extraction. The powder compounds will facilitate the insertion into processed products, such as meat products or packaging, acting as a natural antioxidant able to inhibit lipidic and protein oxidation reactions.

## Figures and Tables

**Table 1:** Results of antioxidant activity of coffee husk extracts.

Extraction Method	Extract	%DPPH Inhibition	%ABTS Inhibition	FRAP (μmol TE/g)
Water bath	EBE	14.17 ± 0.04	52.56 ± 0.09	**1291.85± 0.02**
	EBW	37.52 ± 0.1	76.05 ± 0.6	2103.9 ± 0.8
	EBM	67.52 ± 0.1	91.48 ± 0.1	**2639.4 ± 0.02**
	EBDE	15.86 ± 0.3	56.09 ± 0.8	**2425.9 ± 0.1**
	EBDW	72.50 ± 0.1	**81.63 ± 0.01**	58.73 ± 0.02
	EBDM	**84.95 ± 0.02**	**92.81 ± 0.01**	23.38 ± 0.1
Ultrasound	EUE	23.16 ± 0.01	48.75 ± 0.4	30.01 ± 0.9
	EUW	19.55 ± 0.01	51.97 ± 0.3	51.01 ± 0.6
	EUM	13.24 ± 0.2	64.03 ± 0.3	64.11 ± 0.02
	EUDE	2.44 ± 0.1	51.24 ± 0.7	**3136.4 ± 0.1**
	EUDW	53.71 ± 0.02	**93.24 ± 0.01**	89.75 ± 0.02
	EUDM	**84.20 ± 0.03**	**97.21 ± 0.01**	27.62 ± 0.9

TE: Trolox equivalente.

**Table 2:** Total phenolic content, total flavonoids, and condensed tannins extracted from coffee husk.

Extraction Method	Extract	Total Phenolic (mg CAE/g)	Total Flavonoid (mg CE/g)	Condensed Tannins (mg CE/g)
Water bath	EBE	31.35 ± 1.90	0.79 ± 0.12	13.50 ± 1.8
	EBW	42.51 ± 0.72	0.63 ± 0.07	17.43 ± 0.7
	EBM	95.00 ± 1.39	2.81 ± 0.12	37.07 ± 1.09
	EBDE	45.20 ± 1.90	4.1 ± 0.5	15.12 ± 0.8
	EBDW	47.57 ± 0.44	**9.93 ± 0.9**	35.92 ± 0.4
	EBDM	**97.89 ± 0.72**	**4.53 ± 1**	**79.71 ± 0.8**
Ultrasound	EUE	16.54 ± 2.18	0.75 ± 0.42	12.73 ± 0.1
	EUW	34.10 ± 3.78	0.21 ± 0.16	17.04 ± 4
	EUM	77.57 ± 0.44	2.00 ± 0.19	23.75 ± 8
	EUDE	26.74 ± 2.99	5.16 ± 0.3	13.21 ± 0.4
	EUDW	36.16 ± 3.26	4.61 ± 0.2	32.04 ± 1.30
	EUDM	**90.95 ± 1.73**	**15.69 ± 0.3**	**72.53 ± 1.01**

CAE: Chlorogenic acid equivalente; CE: Catechin equivalente.

**Table 3:** Profile of major phenolic compounds from coffee husk extracts.

Extraction Method	Extract	Gallic Acid (µg/g) R_t_ = 4.67 min	Chlorogenic Acid (µg/g) R_t_ = 6.03 min	Caffeic Acid (µg/g) R_t_ = 7.28 min
Water bath	EBE	3.18 ± 0.04	29.35 ± 2.52	1.61 ± 0.35
	EBW	4.45 ± 0.44	19.64 ± 3.30	1.32 ± 0.26
	EBM	2.17 ± 0.07	25.93 ± 0.84	1.40 ± 0.10
	EBDE	Nd	67.49 ± 1.01	1.27 ± 0.01
	EBDW	60.22 ± 1.95	220.89 ± 12.51	2.76 ± 0.49
	EBDM	20.81 ± 4.53	337.07 ± 9.88	6.15 ± 0.60
Ultrasound	EUE	0.72 ± 0.22	24.78 ± 2.86	1.38 ± 0.11
	EUW	1.91 ± 0.15	27.99 ± 0.55	1.23 ± 0.15
	EUM	2.41 ± 0.77	27.64 ± 1.68	1.95 ± 0.34
	EUDE	Nd	37.29 ± 1.35	1.19 ± 0.04
	EUDW	33.15 ± 4.15	178.68 ± 7.56	3.35 ± 0.02
	EUDM	25.52 ± 4.58	304.36 ± 13.01	4.53 ± 1.04

R_t_ = retention time (min).
